# Lessons Learned about Pneumonic Plague Diagnosis from 2 Outbreaks, Democratic Republic of the Congo

**DOI:** 10.3201/eid1705.100029

**Published:** 2011-05

**Authors:** Eric Bertherat, Philippe Thullier, Jean Christophe Shako, Kathleen England, Mamadou-Lamine Koné, Lorraine Arntzen, Herbert Tomaso, Louis Koyange, Pierre Formenty, Florent Ekwanzala, Rosa Crestani, Isa Ciglenecki, Lila Rahalison

**Affiliations:** Author affiliations: World Health Organization, Geneva, Switzerland (E. Bertherat, P. Formenty);; Centre de Recherche du Service de Santé des Armées, Grenoble, France (P. Thullier);; Plague Reference Laboratory, Bunia, Democratic Republic of the Congo (J.C. Shako);; National Institutes of Health, Bethesda, Maryland, USA (K. England);; World Health Organization, Brazzaville, Republic of Congo (M.-L. Koné);; National Health Laboratory Service, Johannesburg, South Africa (L. Arntzen);; Bundeswehr Institute of Microbiology, Munich, Germany (H. Tomaso);; Institut National de la Recherche Biomédicale, Kinshasa, Democratic Republic of the Congo (L. Koyange);; World Health Organization, Kinshasa (F. Ekwanzala);; Médecins Sans Frontière, Bruxelles, Belgium (R. Crestani);; Médecins sans Frontières, Geneva (I. Ciglenecki);; Institut Pasteur, Antananarivo, Madagascar (L. Rahalison)

**Keywords:** bacteria, Yersinia pestis, plague, pneumonic plague, outbreak response, bacteria, rapid diagnosis, Democratic Republic of the Congo, synopsis

## MEDSCAPE CME

Medscape, LLC is pleased to provide online continuing medical education (CME) for this journal article, allowing clinicians the opportunity to earn CME credit.

This activity has been planned and implemented in accordance with the Essential Areas and policies of the Accreditation Council for Continuing Medical Education through the joint sponsorship of Medscape, LLC and Emerging Infectious Diseases. Medscape, LLC is accredited by the ACCME to provide continuing medical education for physicians.

Medscape, LLC designates this Journal-based CME activity for a maximum of 1 *AMA PRA Category 1 Credit(s)*^TM^. Physicians should claim only the credit commensurate with the extent of their participation in the activity.

All other clinicians completing this activity will be issued a certificate of participation. To participate in this journal CME activity: (1) review the learning objectives and author disclosures; (2) study the education content; (3) take the post-test and/or complete the evaluation at www.medscape.org/journal/eid; (4) view/print certificate.

**Release date: April 25, 2011; Expiration date: April 25, 2012**

## Learning Objectives

Upon completion of this activity, participants will be able to:

- Describe transmission, presentation, and course of pneumonic plague
- Describe how outbreaks of pneumonic plague in the Democratic Republic of the Congo were recognized
- Describe frontline response strategy to pneumonic plague that will facilitate implementation of the first control measures

## MEDSCAPE CEM EDITOR

**Nancy Mannikko, **Technical Writer/Editor, *Emerging Infectious Diseases. Disclosure: Nancy Mannikko has disclosed no relevant financial relationships.*

## MEDSCAPE CME AUTHOR

**Laurie Barclay, MD, **freelance writer and reviewer, Medscape, LLC. *Disclosure: Laurie Barclay, MD, has disclosed no relevant financial relationships.*

## AUTHORS

*Disclosures:* ***Eric Bertherat, MD; Philippe Thullier, MD; Jean Christophe Shako; Kathleen England; Mamadou-Lamine Koné, MD; Lorraine Arntzen; Herbert Tomaso, MD; Louis Koyange, MD; Pierre Formenty, MD; Florent Ekwanzala, MD; Rosa Crestani, MD; Isa Ciglenecki, MD;*** *and* ***Lila Rahalison, PhD,*** *have disclosed no relevant financial relationships.*
