# Supplementary material for: Lessons Learned about Pneumonic Plague Diagnosis from 2 Outbreaks, Democratic Republic of the Congo
Source: Emerg Infect Dis. 2011 May;17(5):778–84. doi: 10.3201/eid1705.100029 (PMC3321750; doi:10.3201/eid1705.100029)
Supplement: Related texts

# Review article: Lessons Learned about Pneumonic Plague Diagnosis from 2 Outbreaks, Democratic Republic of the Congo

**DOI:** 10.3201/eid1705.100029

**Authors:** Eric Bertherat, Philippe Thullier, Jean Christophe Shako, Kathleen England, Mamadou-Lamine Koné, Lorraine Arntzen, Herbert Tomaso, Louis Koyange, Pierre Formenty, Florent Ekwanzala, Rosa Crestani, Isa Ciglenecki, Lila Rahalison

**Affiliations:** Author affiliations: World Health Organization, Geneva, Switzerland (E. Bertherat, P. Formenty);; Centre de Recherche du Service de Santé des Armées, Grenoble, France (P. Thullier);; Plague Reference Laboratory, Bunia, Democratic Republic of the Congo (J.C. Shako);; National Institutes of Health, Bethesda, Maryland, USA (K. England);; World Health Organization, Brazzaville, Republic of Congo (M.-L. Koné);; National Health Laboratory Service, Johannesburg, South Africa (L. Arntzen);; Bundeswehr Institute of Microbiology, Munich, Germany (H. Tomaso);; Institut National de la Recherche Biomédicale, Kinshasa, Democratic Republic of the Congo (L. Koyange);; World Health Organization, Kinshasa (F. Ekwanzala);; Médecins Sans Frontière, Bruxelles, Belgium (R. Crestani);; Médecins sans Frontières, Geneva (I. Ciglenecki);; Institut Pasteur, Antananarivo, Madagascar (L. Rahalison)

## Abstract

Pneumonic plague is a highly transmissible infectious disease for which fatality rates can be high if untreated; it is considered extremely lethal. Without prompt diagnosis and treatment, disease management can be problematic. In the Democratic Republic of the Congo, 2 outbreaks of pneumonic plague occurred during 2005 and 2006. In 2005, because of limitations in laboratory capabilities, etiology was confirmed only through retrospective serologic studies. This prompted modifications in diagnostic strategies, resulting in isolation of *Yersinia pestis* during the second outbreak. Results from these outbreaks demonstrate the utility of a rapid diagnostic test detecting F1 antigen for initial diagnosis and public health management, as well as the need for specialized sampling kits and trained personnel for quality specimen collection and appropriate specimen handling and preservation for plague confirmation and *Y. pestis* isolation. Efficient frontline management and a streamlined diagnostic strategy are essential for confirming plague, especially in remote areas.

Plague is a zoonotic disease caused by the bacterium *Yersinia pestis,* an agent circulating among small mammals and fleas (*1**–**3*). Human infection is usually transmitted by the bite of an infected flea, and bubonic plague is the most common form of the disease. The illness can progress into advanced clinical forms of septicemic and pneumonic plague. Pneumonic plague is of serious concern because of the potential for human-to-human transmission from aerosolized bacteria spread through coughing. Pneumonic plague can lead to localized outbreaks, or even devastating epidemics, because the infectious dose by inhalation can be as low as 100–500 organisms (*4*). Untreated, pneumonic plague usually leads to death within 2–4 days after respiratory exposure; in some instances, death occurs as rapidly as 24 hours after exposure (*1**,**3**,**5**,**6*). The rapid onset and high lethality are its only distinguishable clinical features as the disease otherwise manifests itself as a severe respiratory infection that could be caused by various pathogens.

In regions where clinicians are unfamiliar with plague, risk of misdiagnosis is high, and specific diagnostic tools are often not readily accessible in remote areas. Identification of the causal agent is critical for implementing immediate public health measures in the community. Furthermore, in previously plague-free areas, confirmation of the diagnosis may lead to the identification of the emergence of a new natural focus that requires a revised public health strategy.

Rapid diagnostic tests (RDTs) are available and can be effective for helping manage such situations; however, they do not replace bacterial isolation, which remains the most accurate method and enables crucial antimicrobial drug susceptibility testing. In the absence of bacterial isolation, plague confirmation requires serologic detection of a 4-fold rise in plague-specific antibodies, or, in plague-endemic regions, a positive RDT (*7*). Whichever technique is used, plague confirmation depends on appropriate sample collection, effective sample preservation measures, and direct transportation of samples to diagnostic laboratory facilities, as illustrated in the 2 pneumonic plague outbreaks we discuss. We highlight important lessons on frontline sample collection methods and transport and provide an awareness of the challenges faced in regard to diagnostic strategies in remote, war-torn regions of the world.

## The Outbreaks

Plague remains a concern in several countries, particularly in Africa (*8**–**12*). The Democratic Republic of the Congo (DRC), a war-torn country, has the most active focus of plague worldwide. In the northeastern region of Ituri, >1,000 suspected cases are reported each year (*7**,**12**,**13*). In January 2005, an outbreak of highly lethal pneumonia occurred in a diamond mining camp in a remote area of the Oriental Province (Figure 1), 25 km from the village of Zobia, Bas-Uele (*13**,**14*). Clinical signs and fast spread of the disease lead to the suspicion of pneumonic plague. No previous cases of plague had been reported in this region, which has a history of severe security problems and where at the time of the outbreak a United Nations peacekeeping operation was ongoing. In August 2006, an outbreak of a similar nature occurred in a gold mining camp 200 km from the Zobia camp, near Bolebole, Haut Uele (*15*).

**Figure 1:** Transport routes of clinical specimens from 2 pneumonic plague outbreaks, Democratic Republic of the Congo, 2005 and 2006.

### Investigative Plans

At the request of Congolese officials, World Health Organization (WHO) response teams intervened in both outbreaks. However, because of a delayed alert and bureaucratic challenges, the teams arrived 2 months after onset of the epidemics, which limited the number of case-patients who were available for clinical examination and appropriate sampling. As a result of the circumstances, the following investigative plan was established.

The suspected plague deaths that occurred before the WHO response team arrival were attributed to plague based on the epidemiologic context, the reported clinical signs, and the rapid disease onset typically observed for pneumonic plague. However, no postmortem specimens could be taken for confirmation. Suspected convalescents were tracked, interviewed, and blood samples were collected in standard serologic tubes by a clinician and stored at 4°C for retrospective serologic testing.

Each new patient with clinically suspected pneumonic plague was admitted to an isolation center to receive the first doses of the national protocol treatment. Blood was immediately sampled and sputum samples were collected by a trained biologist from the National Plague Reference Laboratory who supported the WHO mission. Sampling kits, designed by the Institut Pasteur de Madagascar (IPM; Antananarivo, Madagascar) for improving laboratory confirmation of plague cases in this country, were used to collect and prepare sputum samples for transport. The kits contained the necessary equipment for sampling either sputum or bubo aspirate and provided an effective method for collecting pure samples with minimal chance of contamination. One kit was required per patient; each contained a sterile syringe for sample extraction from a bubo or sputum sampling cup, an Eppendorf tube containing 1 mL of phosphate-buffered saline (PBS) for sample dilution, a culture tube with Cary Blair (CB) medium, a sterile swab for placement of pure sample into CB media for transport, a sterile test tube for RDT, isopropyl alcohol and antiseptic towelettes, and a patient record form. Safety procedures included use of proper personal protective equipment and appropriate waste management.

### Specimen Handling and Transportation

In the 2005 Zobia outbreak, handling and transportation of the specimens were planned as follows. Sputum specimens were immediately evaluated on site by a biologist of the National Plague Reference Laboratory using direct microscopy and RDT. The specimens were then transported to a temporary second-line regional laboratory in Kisangani, DRC, by air (2 hours) on roughly a weekly basis. In Kisangani, a biologist from IPM who was assigned to the WHO mission performed culture identification for *Y. pestis* and direct microscopy and prepared specimens for the twice weekly air shipment (>1,000 km) to the Institut National pour la Recherche Biologique in Kinshasa, DRC. At the time of arrival at Institut National pour la Recherche Biologique, specimens were further distributed by commercial air carriers to IPM in Antananarivo, Madagascar; the National Institute for Communicable Diseases (NICD) in Johannesburg, South Africa; and the Centre de Recherche du Service de Santé des Armées in Grenoble, France, for additional analyses and serologic testing (Figure 2, panel A).

**Figure 2:** Flow of sample processing for specimens for pneumonic plague outbreaks in Zobia, Democratic Republic of the Congo (DRC), 2005 (A), and Bolebole, DRC, 2006 (B). PBS, phosphate-buffered saline; RDT, rapid diagnostic test; RT, reverse transcription; CB, Cary Blair; BHI/CIN, brain–heart infusion; cefsulodin-Irgasan-novobiocin; INRB, Institut National pour la Recherche Biologique; IPM/NICD/CRSSA, Institut Pasteur de Madagascar/National Institute for Communicable Diseases/Centre de Recherche du Service de Santé des Armées; DFA, direct fluorescent antibody.

In the 2006 Bolebole outbreak, a streamlined strategy was defined to limit sample passages and delays in transport. Emphasis was placed on RDT and direct microscopy on site, but no other manipulation of samples was allowed. Regional and national steps were bypassed and samples were sent directly to the international reference laboratory at IPM, where confirmatory tests were performed (Figure 2, panel B).

### Specimen Analyses

Sputum smears were stained (Gram and Wayson) and directly examined by microscopy according to standard protocols (*16*). In addition, sputum was extracted from swabs stored in CB by using 1 mL of PBS and cultured by using standard methods for plague isolation as recommended by WHO (*16*). Suspect colonies were confirmed by using standard biochemical tests and phage lysis as described (*16**–**19*). The above methods were performed at Kisangani, NICD, and IPM during the first outbreak and only at IPM during the second outbreak.

RDT for *Y. pestis* specific F1-antigen detection by immunochromatography was performed at the frontline. The dipstick is contained in an individually vacuum-sealed package with desiccant to maintain stability and sterility and is used with the sampling kit described above. The specimen was processed by using 0.5 mL of thick sputum diluted in 1 mL of PBS and homogenized by using a sterile syringe. A 200-μL aliquot of homogenate was placed in a sterile test tube and the dipstick applied. The test was considered positive when 2 pink lines (control line + test line) appeared after 15 minutes as described (*18*). To simplify the procedures in the field, semiquantitative grading was not requested.

One serum sample was drawn as early as possible after onset of symptoms and a second serum sample 10 days later to allow for the development of an antibody response. A positive result was determined by a 4-fold increase in the antibody titer from the first to the second sample (*1*). Serum was transported at 4°C or at ambient temperature and filter-sterilized upon arrival. ELISA detecting anti-F1 immunoglobulin (Ig) G was performed as previously described (*20*) at Centre de Recherche du Service de Santé des Armées.

Direct fluorescent antibody (DFA) staining was performed at NICD on sputum specimens from the first outbreak according to standard protocols (*16**,**21**,**22*). Smears were a thin impression (touch preparation) of sputum on swabs transported in CB medium. The presence of plague bacilli was tested through the direct binding of fluorescein isothiocyanate–conjugated F1 antibody to antigen. A smear was positive when bright, intense green staining around bacteria was observed.

## Field Implementation

The first plague outbreak (Zobia) took place between December 15, 2004, and March 11, 2005, and resulted in 130 cases, all pneumonic except for 2 septicemic, and 57 deaths (44% fatality rate). Forty-eight deaths had already occurred. No specimens had been taken when the WHO response team arrived in the field on February 19. At the time of arrival, specimens were collected from 46 patients with acute illness (2 had a pair of blood samples collected), and from 41 convalescents (4 had a pair of blood samples collected) (Table). *Y. pestis* was not isolated from any specimen from a patient with acute illness, but samples from 5 of the 6 patients with paired blood samples converted. Of note, 18 of 37 (49%) of sputum specimens from patients with acute illness tested by RDT were positive.

**Table:** Laboratory results for 87 and 117 suspected cases of pneumonic plague during outbreaks in 2005 and 2006, respectively, Democratic Republic of Congo*

Location, year	No. cases for which samples were collected from patients	No. positive samples/no. tested (% positive)					
		RDT	Microscopy	*Yersinia pestis* isolation	DFA	PCR	IgG
Zobia, 2005†	Acute, 44	17/35 (48.6)	31/44 (70.4)	0/44	6/43 (13.9)	2/42 (4.8)	7/18 (3.9)
	Paired samples, 6	1/2 (50)	1/2 (50)	NA	0/2	0/2	6//12 (50.0)
	Convalescent, 37	NA	NA	NA	NA	NA	12/37 (3.2)
Bole, 2006‡	Acute, 98	23/96 (24.0)	38/67 (56.7)	4/96 (4.2)	NA	NA	2/89 (2.2)
	Convalescent, 19	NA	NA	NA	NA	NA	0/19 (0)

*RDT, rapid diagnostic test; DFA, direct fluorescent antibody test; PCR, real-time PCR; Ig, immunoglobulin; NA, not applicable. †Of 130 cases identified, samples were collected from 87 patients. ‡Of 162 cases identified, samples were collected from 117 patients.

The second outbreak (Bolebole) lasted from August 21 through October 21, 2006. In a preliminary investigation, 15 sputum samples were collected. However, these samples were stored and transported in dry containers and did not yield results indicative of plague when they were analyzed in a reference laboratory. A second investigation in cooperation with the National Plague Reference Laboratory suspected 5 other patients were infected. Forty-five deaths were reported. RDTs were performed in the field and 2 immediately showed positive results, prompting a new request for an international intervention, which led to the third investigation. During this phase, specimens were collected from 98 patients having acute illness (of which 2 specimens were excluded) and 19 convalescents; no paired sera were collected (Table). *Y. pestis* was isolated and confirmed by its biochemical profile from 4 sputum specimens. Of note, 23 of 96 (24%), including all specimens in which *Y. pestis* was isolated, were positive by RDT. Local authorities first reported 1,597 suspected pneumonic cases and 54 deaths. However, the international mission observed a significant overreporting caused by inappropriate case definition. Most of the medical files were not able to be retrieved, but the number of cases was estimated at 162 on the basis of information included in the registries. We can retrospectively describe the 2 outbreaks according to the WHO case definition as follows. In Zobia, in the absence of the isolation of *Y. pestis*, 5 cases were confirmed (seroconversion), 10 were probable (>2 positive tests), and 115 remained suspect. In Bolebole, 23 cases were confirmed (4 isolations plus 19 positive RDT), 22 were probable (direct examination or single serology positive), and 117 remained suspect.

### Specimen Handling and Transport

Pneumonic plague was suspected late after the onset of the outbreak in Zobia. No clinical specimen had been taken before the arrival of the WHO response team because first-line staff had not been properly trained and appropriate sampling materials were not available. After deployment, and under direct management of the response team, the frontline laboratory conducted RDT and direct microscopy; results indicated plague. All patients with suspected plague were subsequently isolated and treated, and close contacts received chemoprophylaxis as recommended by WHO (*1*). During this outbreak, difficulties with isolating *Y. pestis* were anticipated and a second-line laboratory in Kisangani was established. Unfortunately, the functions of this laboratory were limited by power and water shortages, which restricted the capacity to perform. As such, this laboratory in practice served only as a logistic platform for the air shipment of specimens to Kinshasa. Shipments from Zobia to Madagascar took 8–40 days (median 18.5 days). Sputum samples in CB were stored at 28°C–30°C, and serum was stored at 4°C.

At Bolebole, greater emphasis was placed on front-line sampling, avoiding dilutions, and multiple handling of specimens. Only direct microbiology and RDT for F1 detection were conducted on site, which provided results indicating plague. Samples were then obtained by specimen collection kits and sent in good condition. Samples were stored at the same temperatures as during the first outbreak, were transported once a week by road from Bolebole to Isiro (2 days), and then shipped by air to Kinshasa on an 8-hour flight with 3 intermediate landings. Finally, specimens were shipped to Madagascar weekly by commercial airlines. In total, the transport of samples from Bolebole to IPM took 4–5 days.

### Use of RDT

Cost-effective, easy to use, and reliable, the RDT can be considered as an alert tool. It has proven to be rapid and easy to use at the bedside with 100% specificity and sensitivity (*18*). Importantly, detection of the F1-antigen is possible even in cases that have been previously treated, which was common for many of the patients in the outbreaks we describe. However, the RDT is not a screening test and must be used only to confirm a clinical suspicion. The RDT is also helpful for outbreak control. In both Zobia and Bolebole, one of the most challenging and time-consuming activities was managing the close contacts (≈25 for each suspected case). Even though the RDT results have no confirmatory value during the early phase of the outbreak, they enable management of close contacts to be focused around cases defined as probable within 15 minutes. Notably, the RDT becomes a field confirmation test after *Y. pestis* has been identified on site (*7*).

### Specimen Collection and Testing

In addition to sputum samples, a serum sample was drawn from all patients admitted to the isolation center as well as from convalescents. However, in circumstances as described above, it was challenging to obtain a second blood sample from the patients 10 days later. As for the suspect cases that occurred before the arrival of the response team, motivation for testing was weak and no suspect patients returned. This was because most patients were young male adults, who returned to mining activities after cure and release from the isolation center. Any kind of control was perceived only as a limitation to business as usual. Overall, only 6 pairs of serum samples were obtained from patients treated at an early stage in Zobia, despite a strong emphasis being placed on serology for confirmation. The situation was worse in Bolebole, as no paired serum samples were obtained.

Isolation of *Y. pestis* from sputum was desired but challenging because of administration of antimicrobial drugs to patients before specimen collection, the nonsterile nature of the specimen, the slow growth of *Y. pestis* in culture, and prolonged specimen handling and transport. In addition, results from DFA and real-time PCR testing may also have been affected by prolonged handling and transport.

Because of the limitations in the field, it is difficult to extract information in regard to the respective performances of different diagnostic techniques. RDT is an established test for Africa and is known for its high sensitivity and specificity. If nearly half of the tested patients were positive in Zobia and 24% in Bolebole (Table), this can be due to inappropriate specimens (saliva instead of sputum) or a misdiagnosis. However it should be noted that 4 of 5 patients with confirmed plague in Zobia had a positive RDT result (DFA analyses were positive for 2 of them). In Bolebole, the 4 patients with plague confirmed by culture also had a positive RDT result.

### Scope of the Outbreak

The primary objective of the laboratory investigation was to confirm the existence of ongoing pneumonic plague in the communities, to set up an ad hoc control strategy, and to stop the spread of disease. With regard to the magnitude of the 2 outbreaks, one cannot discard the possibility that a certain number of reported cases were not due to plague. However, one cannot discard the possibility that several plague cases were neither diagnosed nor reported. This is particularly true for the period that preceded the international intervention. In Zobia, to be as specific as possible, only the patients who died of a short-course and severe febrile pneumonic syndrome at this period were considered to have died of plague. But what was the number of survivors? This is difficult to project. The case-fatality ratio observed during the mission (11.0%) cannot be applied at the preintervention period because case management had greatly improved. In addition, the peak of the outbreak had passed, and the number of true plague cases among those clinically detected was probably lower because less than half of the patients with acute illness had a positive RDT (Table). The situation was similar at Bolebole.

## Strategy for Sub-Saharan Africa

Rural Africa reports >95% of all plague cases worldwide. The cases usually occur in areas where the disease is endemic, but emergence is not exceptional. In plague-endemic endemic areas, priority must be given to reinforcement of the local diagnostic capacities. Sustainable and simple operating procedures should be promoted, and specimens should be treated locally or regionally as much as possible, avoiding challenging transport issues. Specimen collection kits and RDT must be provided for first-line detection and collection. Promoting RDT use, however, is much more than prepositioning dipsticks. RDT use must include training, supervision, and the necessary materials to appropriately collect and process the samples. Efforts must be given priority in districts with endemic plague and neighboring ones. Therefore, RDT should be part of a global strategy to reinforce the surveillance and control capacities, including laboratory diagnosis, at the district or provincial level. As shown in DRC, a provincial-level laboratory can perform biochemical identification from culture, confirm RDT results, and supervise RDT use in the framework of a plague surveillance and control strategy.

In previously plague-free areas, the approach is different. Any emergence is considered a public health emergency of international concern to be reported to WHO. Such situation requires a national or international response, which must include professionally trained and equipped teams to appropriately manage the outbreak and direct connections to an international reference laboratory. Specimen collection kits and RDT are efficient and easy-to-use tools for the investigation and the first control measures. However, the absolute need to demonstrate the etiology of the outbreak justifies any additional efforts that human and technical resources specifically deployed allow. Even if only a seroconversion is confirmatory, a unique seropositivity is considered an interesting finding in an area where *Y. pestis* is presumed not to circulate. The practical difficulties experienced in Zobia and Bolebole in collecting paired blood samples were linked particularly to a chaotic context. In a more relaxed situation, collecting paired samples must be a priority because quality sputum specimens will remain difficult to obtain in the field. For the same reason, blood cultures could be attempted. This would require specific transport media, but this is not an issue for a national or international intervention. To date, according to the WHO case definition, neither PCR nor DFA is established as a confirmatory test. Both techniques have limitations and can be affected by the specimen conditions or a prior administration of antimicrobial drugs. However, positive results can reinforce the suspicion and contribute to the understanding of the outbreak. Both should be considered an important part of the biological investigation when plague emergence is suspected.

## Conclusions

Pneumonic plague emerged in new locations in the Oriental Province of DRC with outbreaks in 2005 and 2006. Due to the remoteness and political instability in this region, access to these locations was constrained, which added to the challenges in managing these outbreaks and confirming their cause. Compared with the first outbreak, the second outbreak showed that the detection of the F1-antigen by RDT was able to strengthen the frontline response strategy. In this experience, the RDT test proved to be an important step for preliminary diagnosis, which led to an early alert of the outbreak and facilitated the implementation of the first control measures.

However, in previously unknown plague foci, the isolation of *Y. pestis* remains the absolute proof of the outbreak cause. The importance of a well-prepared and well-equipped strategy was demonstrated in 2006. It can be summarized as an empowered frontline and a robust reference laboratory with efficient transportation and minimized handling between the two. In 2005 and 2006, an international reference laboratory was essential. Today, in similar situations of emergence of new plague foci, strengthening the national plague reference laboratory would likely limit the need for international transportation of potentially dangerous specimens to only the first confirmations. This effort should be extended to all the plague-endemic countries in Africa.

## References

[1] Dennis DT, Gage KL, Gratz N, Poland JD, Tikhomirov E. Plague manual: epidemiology, distribution, surveillance and control. Geneva: World Health Organization; 1999.

[2] Perry RD, Fetherston JD. *Yersinia pestis*—etiologic agent of plague. Clin Microbiol Rev. 1997;10:35–66.899385810.1128/cmr.10.1.35PMC172914

[3] Pollitzer R. Plague. Geneva: World Health Organization; 1954. p. 233–50.

[4] Franz DR, Jahrling PB, Friedlander AM, McClain DJ, Hoover DL, Bryne WR, Clinical recognition and management of patients exposed to biological warfare agents. JAMA. 1997;278:399–411. 10.1001/jama.278.5.3999244332

[5] Inglesby TV, Dennis DT, Henderson DA, Bartlet JG, Ascher MS, Eitzen E, Plague as a biological weapon: medical and public health management. JAMA. 2000;283:2281–90. 10.1001/jama.283.17.228110807389

[6] McCrumb FR, Mercier S, Robic J, Bouillat M, Smadel JE, Woodward TE, Chloramphenicol and terramycin in the treatment of pneumonic plague. Am J Med. 1953;14:284–93. 10.1016/0002-9343(53)90040-013030486

[7] World Health Organization. International meeting on preventing and controlling plague: the old calamity still has a future. Wkly Epidemiol Rec. 2006; 278–84.16841399

[8] Stenseth NC, Atshabar BB, Begon M, Belmain SR, Bertherat E, Carniel E, Plague: past, present, and future. PLoS Med. 2008;5:e3. 10.1371/journal.pmed.005000318198939PMC2194748

[9] Begier EM, Asiki G, Anywaine Z, Yockey B, Schriefer ME, Aleti P, Pneumonic plague cluster, Uganda, 2004. Emerg Infect Dis. 2006;12:460–7.1670478510.3201/eid1203.051051PMC3291454

[10] Cot S, Bertherat E. Bubonic plague: the old scourge is alive and well. Inter-regional Conference on Prevention and Control of Bubonic Plague, April 7–11, 2006, Antananarivo, Madagascar. Med Trop (Mars). 2007;67:117–8.17691426

[11] Laudisoit A, Leirs H, Makundi RH, Van Dongen S, Davis S, Neerinchkx S, Plague and the human flea, Tanzania. Emerg Infect Dis. 2007;13:687–93.1755324510.3201/eid1305.061084PMC2738476

[12] World Health Organization. Suspect plague in the Democratic Republic of the Congo. 2006 [cited 2011 Jan 4]. http://www.who.int/csr/don/2006_11_07/en/index.html.

[13] World Health Organization. Interregional Meeting on Prevention and Control of Plague (WHO/HSE/EPR/2008.3). Antananarivo, Madagascar. 2006 [cited 2011 Jan 4]. http://www.who.int/csr/resources/publications/WHO_HSE_EPR_2008_3w.pdf

[14] Bertherat E, Lamine KM, Formenty P, Thullier P, Mondonge V, Mitifu A, Major pulmonary plague outbreak in a mining camp in the Democratic Republic of Congo: brutal awakening of an old scourge [in French]. Med Trop (Mars). 2005;65:511–4.16555508

[15] World Health Organization. Outbreak report: plague, Democratic Republic of the Congo. Wkly Epidemiol Rec. 2006;:397.

[16] Chu M. Laboratory manual of plague diagnostic tests. Geneva: World Health Organization. 2000.

[17] Chanteau S, Ratsitorahina M, Rahalison L, Rasoamanana B, Chan F, Boisier P, Current epidemiology of human plague in Madagascar. Microbes Infect. 2000;2:25–31. 10.1016/S1286-4579(00)00289-610717537

[18] Chanteau S, Rahalison L, Ralafiarisoa L, Foulon J, Ratsitorahina M, Ratsifasoamanana L, Development and testing of a rapid diagnostic test for bubonic and pneumonic plague. Lancet. 2003;361:211–6. 10.1016/S0140-6736(03)12270-212547544

[19] Rasoamanana B, Rahalison L, Raharimanana C, Chanteau S. Comparison of *Yersinia* CIN agar and mouse inoculation assay for the diagnosis of plague. Trans R Soc Trop Med Hyg. 1996;90:651. 10.1016/S0035-9203(96)90420-49015505

[20] Rasoamanana B, Leroy F, Boiser P, Rasolomaharo M, Buchy P, Carniel E, Field evaluation of an immunoglobulin G anti-F1 enzyme-linked immunosorbent assay for serodiagnosis of human plague in Madagascar. Clin Diagn Lab Immunol. 1997;4:587–91.930221010.1128/cdli.4.5.587-591.1997PMC170602

[21] Hudson BW, Quan SF. Use of fluorescent antibody technique for detection of *Pasteurella pestis* in rodents. Trans R Soc Trop Med Hyg. 1960;54:599–600. 10.1016/0035-9203(60)90040-7

[22] Winter CC, Moody MD. Rapid identification of *Pasteurella pestis* with fluorescent antibody: I. Production of specific antiserum with whole cell *Pasteurella pestis* antigen. J Infect Dis. 1959;104:274–80. 10.1093/infdis/104.3.27413654817

# Review article: Lessons Learned about Pneumonic Plague Diagnosis from 2 Outbreaks, Democratic Republic of the Congo

**DOI:** 10.3201/eid1705.100029

**Authors:** Eric Bertherat, Philippe Thullier, Jean Christophe Shako, Kathleen England, Mamadou-Lamine Koné, Lorraine Arntzen, Herbert Tomaso, Louis Koyange, Pierre Formenty, Florent Ekwanzala, Rosa Crestani, Isa Ciglenecki, Lila Rahalison

**Affiliations:** Author affiliations: World Health Organization, Geneva, Switzerland (E. Bertherat, P. Formenty);; Centre de Recherche du Service de Santé des Armées, Grenoble, France (P. Thullier);; Plague Reference Laboratory, Bunia, Democratic Republic of the Congo (J.C. Shako);; National Institutes of Health, Bethesda, Maryland, USA (K. England);; World Health Organization, Brazzaville, Republic of Congo (M.-L. Koné);; National Health Laboratory Service, Johannesburg, South Africa (L. Arntzen);; Bundeswehr Institute of Microbiology, Munich, Germany (H. Tomaso);; Institut National de la Recherche Biomédicale, Kinshasa, Democratic Republic of the Congo (L. Koyange);; World Health Organization, Kinshasa (F. Ekwanzala);; Médecins Sans Frontière, Bruxelles, Belgium (R. Crestani);; Médecins sans Frontières, Geneva (I. Ciglenecki);; Institut Pasteur, Antananarivo, Madagascar (L. Rahalison)

## Earning CME Credit

To obtain credit, you should first read the journal article. After reading the article, you should be able to answer the following, related, multiple-choice questions. To complete the questions and earn continuing medical education (CME) credit, please go to www.medscape.org/journal/eid. Credit cannot be obtained for tests completed on paper, although you may use the worksheet below to keep a record of your answers. You must be a registered user on Medscape.org. If you are not registered on Medscape.org, please click on the New Users: Free Registration link on the left hand side of the website to register. Only one answer is correct for each question. Once you successfully answer all post-test questions you will be able to view and/or print your certificate. For questions regarding the content of this activity, contact the accredited provider, CME@medscape.net. For technical assistance, contact CME@webmd.net. American Medical Association’s Physician’s Recognition Award (AMA PRA) credits are accepted in the US as evidence of participation in CME activities. For further information on this award, please refer to http://www.ama-assn.org/ama/pub/category/2922.html. The AMA has determined that physicians not licensed in the US who participate in this CME activity are eligible for *AMA PRA Category 1 Credits*™. Through agreements that the AMA has made with agencies in some countries, AMA PRA credit is acceptable as evidence of participation in CME activities. If you are not licensed in the US and want to obtain an AMA PRA CME credit, please complete the questions online, print the certificate and present it to your national medical association.

## Article Title: Lessons Learned about Pneumonic Plague Diagnosis from 2 Outbreaks, Democratic Republic of the Congo

## CME Questions

**1. You are a public health official asked to consult in a war-torn region of Africa nonendemic for pneumonic plague. Based on the above report by Dr. Bertherat and colleagues, which of the following observations concerning a new outbreak of respiratory infection would make pneumonic plague most likely?**A. Productive coughB. Gradual progression of symptoms over 1-2 weeksC. Spontaneous recovery in most casesD. High mortality rate within 2–4 days after respiratory exposure**2. Based on the above report, which of the following statements about recent outbreaks of pneumonic plague in the Democratic Republic of the Congo (DRC) is most likely correct?**A. Diagnostic strategies used for the 2005 and 2006 outbreaks were similarB. The Oriental Province where the outbreaks occurred had pre-existing stable infrastructure facilitating diagnosisC. In the second outbreak, detecting the F1-antigen by rapid diagnostic tests (RDT) strengthened the frontline response strategyD. Specialized sampling kits allowed rapid recognition of the first outbreak**3. As a public health official (described in Question 1), which of the following are you most likely to consider for frontline response strategy to pneumonic plague based on the above report?**A. Transporting specimens to distant laboratories with better facilities is preferredB. Specimen collection kits and RDT must be provided for first-line detection and collectionC. RDT is sufficient for surveillanceD. Outbreaks in previously plague-free areas can be managed locally

## Activity Evaluation

**Table:** 

1. The activity supported the learning objectives.				
Strongly Disagree				Strongly Agree
1	2	3	4	5
2. The material was organized clearly for learning to occur.				
Strongly Disagree				Strongly Agree
1	2	3	4	5
3. The content learned from this activity will impact my practice.				
Strongly Disagree				Strongly Agree
1	2	3	4	5
4. The activity was presented objectively and free of commercial bias.				
Strongly Disagree				Strongly Agree
1	2	3	4	5
